# Non-operative treatment versus percutaneous fixation for minimally displaced scaphoid waist fractures in high demand young manual workers

**DOI:** 10.1007/s10195-014-0293-z

**Published:** 2014-04-30

**Authors:** Haroon Majeed

**Affiliations:** Trauma and Orthopaedics, University Hospital of North Staffordshire, Newcastle Road, Stoke-on-Trent, ST4 6QB England, UK

**Keywords:** Minimally displaced scaphoid fractures, Percutaneous fixation, Cast treatment, Scaphoid waist fractures

## Abstract

**Background:**

Managing minimally displaced scaphoid fractures in young individuals doing physically demanding work remains an issue of debate due to duration of immobilisation and time required off work. Therefore, early diagnosis and appropriate treatment are important to avoid short- and long-term consequences. The literature lacks the exact definition of minimally displaced scaphoid waist fractures. The objective of this review article was to discuss nonoperative and minimally invasive treatment (percutaneous screw fixation) for minimally displaced scaphoid waist fractures and to systematically review the literature, focussing on young workers with physically demanding employment.

**Materials and methods:**

We searched for articles through the most commonly used portals using appropriate terminologies to identify the most relevant articles in the English language comparing nonoperative and percutaneous fixation methods for these fractures in patients between 16 and 40 years of age. Strict inclusion and exclusion criteria were observed.

**Results:**

Sixty relevant published articles were found. Twenty-one of these were considered valid for inclusion and comprised five randomised controlled trials, three prospective studies, four systematic reviews, three meta-analyses, and six retrospective studies. These studies provided a reasonable account of information on the managing undisplaced and minimally displaced scaphoid waist fractures, with satisfactory clinical and statistical analysis. However, it was difficult to assess the outcomes of minimally displaced fractures in isolation. Furthermore, few of these studies relied on plain radiographs for assessing union and did not report on patients’ work status.

**Conclusion:**

Cast treatment has the disadvantages of longer immobilisation time, joint stiffness, reduced grip strength, and longer time to return to manual work. Percutaneous fixation is aimed at reducing damage to the blood supply and soft tissues, allowing early mobilisation of the wrist and early return to manual work. The best available evidence for percutaneous screw fixation versus cast treatment suggests that percutaneous fixation allows a faster time to union by 5 weeks and an earlier return to manual work by 7 weeks, with similar union rates. This systematic review indicates a potential requirement for a prospective randomised controlled trial to compare these two treatment modalities for minimally displaced scaphoid waist fractures in workers with physically demanding jobs in order to objectively assess functional outcomes, time to union and time to return to work.

**Level of evidence:**

Level 3.

## Introduction

Scaphoid fractures account for 50–80 % of all carpal bone fractures in young and active individuals. Managing scaphoid fractures remains an issue of debate because of the potential risk of delayed union and nonunion. Early diagnosis and appropriate treatment are important in order to avoid avascular necrosis, arthritis and carpal collapse [1]. In young individuals employed in physically demanding work, it is even more challenging because of the duration required for immobilisation and thus time required off work. Managing scaphoid fractures varies among hospitals and depends upon local preferences and protocols. However, as a general principle, management involves balancing risk level based on available evidence [2]. Nonoperative treatment is widely accepted and advocated for acute, undisplaced scaphoid waist fractures [3], and screw fixation (percutaneous or open) has become an acceptable method for treating displaced fractures [4]. How to best manage minimally displaced scaphoid waist fractures remains unclear. Displaced fractures have been described in the literature with fracture gap >1 mm [5], but the exact description of minimally displaced fracture is not available in the literature. Therefore, we consider a minimally displaced fracture as one with <1-mm gap.

Assessing union may be difficult on plain radiographs because the scaphoid is composed of >80 % cartilage and therefore does not develop callus. Radiographic consolidation is often delayed compared with clinical consolidation [6]. The objective of this paper is to discuss nonoperative and minimally invasive treatment (percutaneous screw fixation) for minimally displaced scaphoid waist fractures and systematically review the available literature, keeping the focus on young individuals employed in physically demanding work.

## Materials and methods

Articles were sourced from MEDLINE through and PubMed (1970–2013), Embase (1980–2013), Cochrane controlled trials register electronic databases, Thomson Scientific Web of Science (1993–2013) and Elsevier Scopus. Primary search terms were scaphoid waist fractures *with* minimally displaced *or* cast immobilization *or* plaster *or* minimally invasive surgery *or* percutaneous surgery. All types of studies were included in our initial search and final selection and included randomised controlled trials (RCTs), prospective studies, systematic reviews, meta-analyses, retrospective studies and case-series reviews. Abstracts of the relevant searched articles were screened first to assess their validity for inclusion. If satisfactory, then the full-text articles were obtained through online access or manual search through the library access. Studies included in the final selection reviewed outcomes of nonoperative treatment for minimally displaced fractures or percutaneous fixation techniques or direct comparison of these two modalities of treatment for this specific group of fractures in young individuals. Age criteria were set to include results of patients with mean age between 16 and 40 years. There was no minimum or maximum number of patients in each study. All those studies were excluded, which were based on cadavers, or reported the outcomes of nonunited fractures, or late-diagnosed fractures, or open fixation methods for acute fractures or distal or proximal pole fractures. Duplicate studies were identified and excluded. Studies published only in English or with English translation were selected.

## Results

**Fig. 1:**
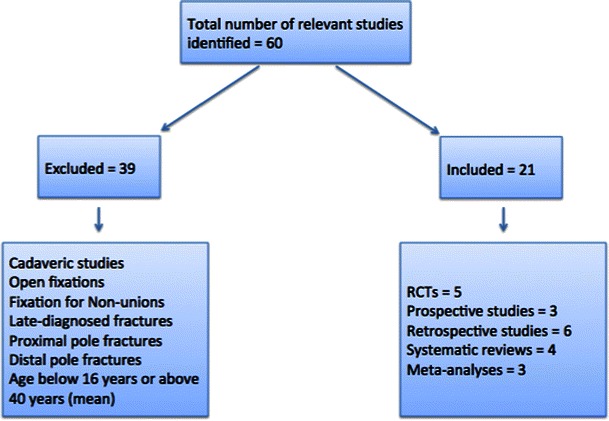
Selection of studies

Based on the above search methodology, 60 relevant published articles were found. Twenty-one were considered valid for inclusion. The selected articles consisted of five randomised controlled trials, three prospective studies, four systematic reviews, three meta-analyses and six retrospective studies (Fig. 1).

Adolfsson et al. [7] compared outcomes of percutaneous fixation (Acutrak screws) with immobilisation in a long scaphoid cast in a randomised controlled trial. Fifty-three patients, mean age 31 years) with undisplaced and minimally displaced acute scaphoid waist fractures were recruited. Fixation group consisted of 25 patients and cast group 28 patients. Cast group was immobilised for 10 weeks; the fixation group was immobilised with a cast for 3 weeks and a removable splint for a further 3 weeks. Computed tomography (CT) scan was used to confirm union in both groups. Results showed a significantly better range of motion (ROM) in the fixation group (*p* < 0.02) but no differences in union rate or grip strength.

An RCT by Bond et al. [8] compared percutaneous fixation (Acutrak screws) with immobilisation in a long scaphoid cast. Twenty-five military personnel with acute undisplaced and minimally displaced scaphoid waist fractures were recruited, with an average age of 24 years. The fixation group had 11 and the cast group 14 patients. The cast group was immobilised in a long cast for 6 weeks, followed by a short cast until union was achieved, which was identified clinically and radiographically. The fixation group was immobilised with a short cast for 10 days and a removable splint until union. All patients were followed up for 25 months, and all fractures achieved union. Analysis showed significant reduction in time to union in the fixation group (7 vs. 12 weeks, *p* = 0.0003) and time to return to full duty in the fixation group (8 vs. 15 weeks, *p* = 0.0001). McQueen et al. [9], in their RCT, compared percutaneous fixation (Acutrak screws) with immobilisation in a Colles’ cast, with the thumb out of cast. Sixty patients with acute undisplaced and minimally displaced scaphoid waist fractures, with a mean age of 27 years, were recruited. Thirty patients were allocated to each group after randomisation. The cast group was immobilised for 8–12 weeks' the fixation group was mobilised immediately with physiotherapy. Clinical and radiological assessments were performed up to 52 weeks, with outcomes being measured by a blinded assessor. Analysis showed a significant reduction in time to union in the fixation group (9.2 vs. 13.9 weeks, *p* < 0.001). There were also significant reductions in the fixation group in times to return to manual work (3.8 vs. 11.4 weeks, *p* < 0.001) and sport (6.4 vs. 15.5 weeks, *p* < 0.001). However, there were no significant differences in functional outcomes or union rates between groups at final follow-up.

Drac and Manak [10] performed a prospective case–control study, with 38 patients in the percutaneous fixation group and 34 in the cast (control) group for acute, undisplaced and minimally displaced waist fractures. Average age was 27 years and minimum follow-up 12 months. Cast group had significantly more nonunions (*p* = 0.024) and restricted ROM and grip strength (*p* < 0.0001). Union was assessed with computed tomography (CT) scan in all patients in the percutaneous fixation group, making union assessment more reliable, but CT was not done for patients in the cast group.

Haddad and Goddard [11] reported 100 % union rate in 50 acute scaphoid waist fractures treated and found an average duration of 55 days after percutaneous screw fixation. Patients had an average age of 26 years. They were allowed full mobilisation immediately after surgery. ROM was equal to contralateral thumb and grip strength (98 %) compared with contralateral thumb at 3 month. All patients returned to manual work within 5 weeks. Brutus and Beaton [12] reported 90 % union rate and good functional results in 30 patients with acute undisplaced scaphoid waist fractures fixed with Herbert screws. Average age was 31 years and average follow-up was 41 months. Average time of cast immobilisation was 3 weeks. ROM was assessed using a goniometer and grip strength using a dynamometer. Inoue and Shionoya, [13], in a prospective study, compared 46 acute scaphoid waist fractures (minimally displaced and undisplaced) treated with percutaneous fixation and 42 fractures treated nonoperatively using below-elbow cast, which included thumb. Average age was 26.5 years. Patients were given the choice of either treatment. Average follow-up was 10 months. The authors found significantly quicker return to manual work (5.8 vs. 10.2 weeks, respectively; *p* < 0.001) and union rate (6 vs. 9.7 weeks, respectively; *p* < 0.001) in the percutaneous fixation group than in the cast group. All fractures united in the percutaneous fixation group; there was one nonunion in the cast group. A similar study by De Vos et al. [14] reported a 97 % union rate after percutaneous fixation of acute scaphoid waist fractures using noncannulated Herbert screws. This series had 44 patients, including 31 heavy manual labourers, with average age of 31 years. Average time to union was 6.4 weeks and return to manual work 41 days. ROM was 97 %, and power grip and pinch grip were 96 % each compared with the contralateral side.

For nonoperative management of acute scaphoid waist fractures, different types of casts are used in routine practice. These include Colles’ cast with wrist in flexion or extension; scaphoid cast below or above elbow; scaphoid cast including or excluding the thumb. Rhemrev et al. [6], in their retrospective study, showed that 81.7 % (58/71) of undisplaced and minimally displaced scaphoid waist fractures achieved clinical and/or radiographic union during 6 weeks of cast immobilisation; another 15.5 % (11/71) required an additional 2 weeks of cast immobilisation. Their overall results showed 97 % union rates within 8 weeks. Clay et al. [5] compared union rates in Colles’ and scaphoid casts in a prospective randomised trial, with 148 patients in the Colles’ cast group and 143 in the scaphoid cast group. No significant difference in union rates were found (*p* = 0.92). Gellman et al. [15] compared long thumb-spica cast with short thumb-spica cast in a prospective randomised controlled trial. They treated 28 patients in long thumb-spica cast for 6 weeks, followed by a short thumb-spica cast for another 6 weeks; 23 patients were treated with short thumb-spica cast throughout the duration of treatment. Significantly shorter time to union was seen (*p* < 0.05) in the long thumb-spica group (9.5 vs. 12.7 weeks). Alho et al. [16] found no significant difference in fracture healing in their prospective study on 100 patients. They compared above- and below-elbow cast immobilisation in a nonrandomised study, with a good number of patients who were alternated in each group. Results of the Gellman et al. and Alho et al. studies should be interpreted with caution because there was significant heterogeneity between them. Retrospective studies by Bongers et al. and Papaloizos et al. [17, 18] favoured operative treatment over cast treatment, reporting improvements in ROM, union rates and return to manual work; however, the studies provide limited quality of evidence due to the nature of the study design.

## Discussion

Traditional cast treatment for minimally displaced scaphoid waist fractures is considered reliable and inexpensive, with low complication rates. Studies show that approximately 85–90 % of these fractures will unite if diagnosed early and treated promptly with cast immobilisation. The main disadvantages of cast treatment are longer immobilisation time, joint stiffness, reduced grip strength and longer time to return to manual work [19]. Immobilisation may be needed for up to 3 months, and patient compliance is thus often unsatisfactory, especially in the presence of low symptom levels, when plasters may be discarded early, resulting in delayed union or nonunion [18, 20]. The advantages of nonoperative treatment have been disputed, and some authors found incomplete healing or nonunion in a high proportion of patients at late follow-up. Cast immobilisation may also lead to ongoing pain and reduced ROM and grip strength [21]. Percutaneous fixation, on the other hand, is aimed at reducing the damage to blood supply and soft tissue, allowing early mobilisation of the wrist and early return to manual work. It can be performed through either the volar or dorsal approach—the former being more popular because of better clinical outcomes, easier access and fewer reported complications [22].

For percutaneous screw fixation, among all studies reviewed, there was a total cohort of 274 patients, with an average age of 27.8 years. Union rate was 98.5 %, with an average time to union of 46 days and average time to return to manual work of 40 days. Among RCTs comparing the two treatment modalities, the RCT performed by Adolfsson et al. [7] was a poor-quality study providing level 2b evidence. They did not report the method of randomisation and lacked power calculation. Furthermore, a paucity of demographic data did not enable a fair comparison between groups; 25 % of patients were lost to follow-up or excluded from analysis, and outcome assessors were not blinded, which may have been a source of bias. However, the RCT reported by Bond et al. [8] was a high-quality study providing level 1b evidence. There were clear inclusion and exclusion criteria, with adequate study power and 100 % follow-up. A limitation of their study was the potential inaccuracy of detecting union with plain radiographs instead of CT scans and the limited times of radiographs being performed. There could be a possibility of observer bias, as two authors themselves assessed the radiographs for union. The study also had poor generalisability, as all participants were full-time military personnel and therefore the times to return to duty may not reflect the time to return to work in the general population. In comparison, the RCT by McQueen et al. [9] was also a high quality study providing level 1b evidence. It is the only study in which the outcomes were measured by a blinded assessor, thereby reducing the risk of bias. A power and sample size calculation was, however, not reported. Rehabilitation may have differed between the groups as not all the patients had physiotherapy. Union rates may be inaccurate as radiographs alone were used to define union due to the reasons discussed earlier.

Among the prospective studies, Drac and Manak [10] performed a well-structured study with satisfactory statistical analysis; however, it was limited by the lack of CT scan comparison in the control (cast) group. In comparison, Haddad and Goddard [11] described systematic methodology and results in their study, but it was limited by the method assessing functional outcome, which was subjective rather than employing a standardised tool; moreover, the type and extent of patient professions were not described. Inoue and Shionoya [13] described good statistical analysis and homogenous groups, but their study was nonrandomised. De Vos et al. [12] performed a retrospective review that had a high likelihood of interobserver variability in measuring ROM and grip strength. Union was assessed on the basis of plain radiographs, making the assessment of union less reliable. Functional assessment was subjective rather than using a standardised tool. Brutus and Beaton reported their results of a retrospective study of results from multiple surgeons, variable follow-up intervals and selection criteria and a 40 % dropout rate due to lost follow-up. These factors reduced the number of patients and the credibility of results due to lack of standardised methodology. It was interesting to note here that different types of screws used in different studies show similar union rates and functional outcomes.

Among studies reporting the outcomes of nonoperative treatment, a review by Rhemrev et al. provided an excellent account with regard to type of cast used, but the study lacked good functional outcome score, and functional outcome assessment was subjective. Some authors have shown that immobilisation in slight dorsal extension has a positive effect on grip strength and wrist joint ROM [5, 15, 23]. The RCT by Clay et al. [5] was a strong study in terms of design and comparable group size, but union rates were assessed on the basis of plain radiographs only. In their series, 13 % (37/291) of patients were reported as having probable union. Although the authors stated that these patients remained asymptomatic at 12 months, there was no later information. Gellman et al. [15] assessed union on plain radiographs in their randomised study between two different types of casts. Their trial was underpowered to support their conclusion of significant benefit of long thumb-spica cast.

This systematic review attempted to focus on a specific group of patients with a specific type of scaphoid fracture. Strict inclusion criteria were observed when selecting the studies, and no limit was applied to the minimum number of patients in each study. Due to lack of a clear definition of minimally displaced fractures, the majority of studies described outcomes of minimally as well as undisplaced fractures. Hence, it was difficult to separately assess the outcomes of minimally displaced fractures. In addition, some studies did not patient profession. In some studies, fracture union was assessed by plain radiographs alone, with no CT scan assessment, hence making accurate assessment of fracture union less reliable [8–10, 13]. 

This systematic review creates a potential requirement for an RCT in order to compare outcomes of nonoperative treatment and percutaneous screw fixation, specifically focusing on minimally displaced scaphoid waist fractures and specifically in young individuals employed in physically demanding work. This will help establish decision-making guidance for clinicians for appropriate management of this group of patients with regards to length of immobilisation and time taken off work.

Managing scaphoid fractures remains a debatable issue because of the potential risk of delayed union and nonunion. Early diagnosis and appropriate treatment are important to avoid long-term consequences associated with nonunion. Managing minimally displaced fractures of the scaphoid waist in young patients employed in physically demanding work is even more challenging due to the issues of functional limitations and time off work. The best available evidence for percutaneous screw fixation versus cast treatment suggests that percutaneous fixation results in a faster time to union by 5 (7 vs. 12) weeks and an earlier return to manual work by 7 (8 vs. 15) weeks, with similar union rates [8, 9]. Cast treatment not only results in longer duration to union but raises concerns of reduced ROM and weakened grip strength. Considering the above evidence, a young worker in a physically demanding job is likely to benefit from percutaneous fixation, which seems to decrease immobilisation time, help achieve full ROM and grip strength and allow earlier return to work. Detailed discussion with the patient is required to explain the pros and cons of each treatment modality. There is a potential requirement for a prospective randomised controlled trial to compare these two treatment modalities for minimally displaced scaphoid waist fractures in workers with physically demanding employment in order to achieve objective assessment of functional outcomes, time to union and return to work.
